# Increasing reproducibility in preclinical stroke research: the correlation of immunofluorescence intensity measurements and Western blot analyses strongly depends on antibody clonality and tissue pre-treatment in a mouse model of focal cerebral ischemia

**DOI:** 10.3389/fncel.2023.1183232

**Published:** 2023-06-02

**Authors:** Anna Prehn, Constance Hobusch, Wolfgang Härtig, Dominik Michalski, Martin Krueger, Bianca Flachmeyer

**Affiliations:** ^1^Institute of Anatomy, Leipzig University, Leipzig, Germany; ^2^Paul Flechsig Institute of Brain Research, Leipzig University, Leipzig, Germany; ^3^Department of Neurology, Leipzig University, Leipzig, Germany

**Keywords:** antibody clonality, protein abundance, degradation products, tissue pre-treatment, neurovascular unit, immunofluorescence labeling, ischemic stroke, paraformaldehyde fixation

## Abstract

In the setting of stroke, ischemia not only impairs neuronal function, but also detrimentally affects the different components of the neurovascular unit, which are shown to be involved in the transition from reversible to long-lasting tissue damage. In this context, the glial proteins myelin basic protein (MBP) and the 2′,3′-cyclic-nucleotide 3′-phosphodiesterase (CNP) as well as the vasculature-associated basement membrane proteins laminin and collagen IV have been identified as ischemia-sensitive elements. However, available data from immunofluorescence and Western blot analyses are often found to be contradictory, which renders interpretation of the respective data rather difficult. Therefore, the present study investigates the impact of tissue pre-treatment and antibody clonality on immunofluorescence measurements of the mentioned proteins in a highly reproducible model of permanent middle cerebral artery occlusion. Here, immunofluorescence labeling using polyclonal antibodies revealed an increased immunofluorescence intensity of MBP, CNP, laminin and collagen IV in ischemic areas, although Western blot analyses did not reveal increased protein levels. Importantly, contrary to polyclonal antibodies, monoclonal ones did not provide increased fluorescence intensities in ischemic areas. Further, we were able to demonstrate that different ways of tissue pre-treatment including paraformaldehyde fixation and antigen retrieval may not only impact on fluorescence intensity measurements in general, but rather one-sidedly affect either ischemic or unaffected tissue. Therefore, immunofluorescence intensity measurements do not necessarily correlate with the actual protein levels, especially in ischemia-affected tissue and should always be complemented by different techniques to enhance reproducibility and to hopefully overcome the translational roadblock from bench to bedside.

## Introduction

Ischemic stroke still represents one of the leading causes of death worldwide (Feigin et al., 2017; Campbell et al., 2019; Virani et al., 2020). Yet, acute therapeutic options are limited to recanalizing treatments such as intravenous thrombolysis and mechanical thrombectomy (Hacke et al., 2008; Goyal et al., 2016). Besides, therapy is not yet available for all the concerned stroke patients and those undergoing recanalization do not always show favorable outcomes (Dirks et al., 2011; Nie et al., 2022; Xiong et al., 2022). It is thus imperative to develop additional neuroprotective therapies, which hopefully lead to a better outcome for stroke patients regarding the mortality or the degree of disability. While stroke research initially rather focused on neuronal damage, it has meanwhile shifted toward a less neurocentric view, considering the whole neurovascular unit (NVU) consisting of the neuronal structures, vascular elements, the extracellular matrix and glial cells (Meairs et al., 2006; Moskowitz et al., 2010; Iadecola and Anrather, 2011; Fisher et al., 2017). In this context, various components of the NVU have been identified to be particularly sensitive to ischemia. Among others, these components comprise the myelin basic protein (MBP) as well as the 2′,3′-cyclic-nucleotide 3′-phosphodiesterase (CNP), both being oligodendroglial proteins and as part of the myelin sheath important for the support, nutrition and insulation of neurons (Sheedlo and Sprinkle, 1985; Bifulco et al., 2002; Nawaz et al., 2013). Other ischemia-sensitive NVU markers are for example laminin and collagen IV, which are both part of the vascular basement membrane (Abrahamson, 1986; Kang and Yao, 2020). However, the literature is not always consistent regarding which alterations specifically occur after an ischemic insult. This diversity is often explained by the heterogeneity of experimental conditions and analysis methods. Nevertheless, there are even studies describing opposite results of an increase of collagen IV or related signals 24 h after an ischemic insult to the brain (Ji and Tsirka, 2012; Härtig et al., 2017; Michalski et al., 2020) while other studies demonstrate a reduction of collagen IV at the same time-point (Hamann et al., 1995, 2002; Vosko et al., 2006; McColl et al., 2008). Similarly controversial reports can be found for other NVU components, including the above mentioned MBP, CNP, and laminin, which renders interpretation of the respective data rather difficult.

Therefore, the present study addresses ischemia-associated changes of MBP, CNP, laminin, and collagen IV to investigate the impact of different methodological aspects including the choice of antibody clonality and tissue pre-treatment used for immunofluorescence labeling in comparison to Western blotting in a mouse model of permanent focal cerebral ischemia.

## Materials and methods

### Animal experiments

Experiments involving animals were reported and performed in accordance with the ARRIVE guidelines, the European Union Directive 2010/63/EU and the German guideline for care and use of laboratory animals after approval by institutional authorities (Landesdirektion Sachsen, Leipzig, Germany).

This study includes brain tissue of mice subjected to 24 h of focal cerebral ischemia using a filament-based model of permanent middle cerebral artery occlusion (MCAO). In total, 16 male C57BL/6J mice with a mean body weight of about 25 g, provided by Charles River Laboratories (Sulzfeld, Germany), were analyzed. Prior to sacrifice, successful infarction as indicated by a neurological deficit (of at least score 2) according to the scoring system of Menzies et al. (1992) was ensured and served as an inclusion criterion. The surgical procedure was conducted in a slightly modified version (Hawkes et al., 2013) of the MCAO technique originally described in Longa et al. (1989). The occlusion was performed using a highly standardized 6-0 silicon-coated filament (Doccol, Sharon, MA, USA), which was advanced into the right internal carotid artery until bending of the filament or any sign of resistance was felt. During the whole MCAO surgery mice underwent anesthesia with 2–2.5% isoflurane (Baxter, Unterschleißheim, Germany) in mixture with 70% N_2_O/30% O_2_. According to the standard operating procedure by Dirnagl and Members of the MCAO-SOP Group (2010), the surgical procedure was kept well below 15 min. To adjust the body temperature to 37°C, a warming pad was thermostatically controlled via a rectal probe. Animals were finally sacrificed 24 h after MCAO induction.

### Tissue processing for immunofluorescence microscopy

Two different ways of tissue processing were used. (a) For the generation of paraformaldehyde-fixed tissue mice were sacrificed and transcardially perfused with saline followed by 4% paraformaldehyde (PFA; Serva, Heidelberg, Germany). The removed brain was preserved in a 4% PFA solution for 24 h. Afterward, the tissue was equilibrated in 30% phosphate-buffered sucrose. Subsequently, forebrains were cut into serial coronal slices of 30 μm thickness using a freezing microtome (Leica SM 2000R, Leica Microsystems, Wetzlar, Germany). For storage, samples were conserved at 4°C in a 0.1 M Tris-buffered saline (TBS) with a pH of 7.4 containing 0.2% sodium azide. (b) For the preparation of fresh-frozen unfixed sections, mice were sacrificed and transcardially perfused with saline, only. Subsequently, the brain was removed and immediately transferred in cryo-embedding medium (Sakura Finetek, Torrance, CA, USA) and snap-frozen in isopentane on dry ice. One hour prior to sacrifice, the animals intravenously received 2 mg fluorescein isothiocyanate (FITC)-albumin (Sigma, Taufkirchen, Germany) dissolved in 0.1 ml saline to demarcate ischemia-affected areas by extravasation of FITC-albumin (Krueger et al., 2019). The brains were coronally sectioned at a thickness of 10 μm on a cryostat (Leica Microsystems), then mounted on fluorescence free-microscope slides and stored at −80°C.

### Immunofluorescence labeling

For immunofluorescence labeling of paraformaldehyde-fixed tissue, free-floating 30 μm forebrain sections were directly rinsed in TBS, while 10 μm thick fresh-frozen unfixed sections were post-fixed with ethanol first, followed by rinsing in TBS. After that, sections were incubated with a blocking solution of 5% normal donkey serum and 0.3% Triton X-100 in TBS for 1 h. Overnight, sections were incubated at 4°C in a solution of primary antibodies, 5% normal donkey serum and 0.3% Triton X-100 in TBS. The antibodies used for this are listed in Table 1. The day after, samples were thoroughly rinsed in TBS and subsequently put into a solution of secondary antibodies diluted in TBS and 2% bovine serum albumin for 1 h at room temperature. After another rinsing step, nuclei were counterstained with 4’,6-diamidino-2-phenylindole (DAPI, Life Technologies, Carlsbad, CA, USA, 1:10,000 in PBS) for 10 min. Finally, sections were rinsed again, free-floating sections were mounted on fluorescence-free microscope slides before sections were coverslipped with fluorescence mounting medium (Dako, Glostrup, Denmark). For selected sets of immunofluorescence labeling as shown in Figure 5, free-floating 30 μm sections were rinsed in TBS, then directly mounted onto microscope slides and treated with antigen retrieval using incubation with 0.05% trypsin in TBS for 15 min at room temperature. Control tissue was mounted on microscope slides and incubated with TBS only. The mounted 30 μm thick sections then underwent the same blocking and incubation steps as described above. For all the applied immunolabeling, the omission of primary antibodies resulted in the absence of labeling (Supplementary Figure 1).

**TABLE 1:** Antibodies used in this study.

	Dilution	Manufacturer
**Immunofluorescence microscopy**		
**Primary antibodies**		
Guinea pig-anti-CNP-1 (polyclonal, 355004)	1:200	Synaptic Systems, Göttingen, Germany
Rat-anti-MBP (monoclonal, ab7349)	1:250	Abcam, Cambridge, United Kingdom
Rabbit-anti-MBP (polyclonal, 295002)	1:400	Synaptic Systems, Göttingen, Germany
Rabbit-anti-Laminin (polyclonal, NB300-144)	1:200	Novus, Littleton, CO, United States
Goat-anti-Collagen-Type IV (polyclonal, AB769)	1:200	Millipore, Burlington, MA, United States
Rabbit-anti-MAP2 (polyclonal, AB5622)	1:200	Sigma Aldrich, Taufkirchen, Germany
Mouse-anti-MAP2 (monoclonal, M4403)	1:200	Sigma Aldrich, Taufkirchen, Germany
**Secondary antibodies**		
AlexaFluor488-donkey-anti-guinea pig (706-545-148)	1:250	Jackson ImmunoResearch, Cambridge, United Kingdom
AlexaFluor488-donkey-anti-rat IgG (A21208)	1:250	Thermo Fisher Scientific, Waltham, MA, United States
AlexaFluor568-donkey-anti-rabbit IgG (A10042)	1:250	Thermo Fisher Scientific, Waltham, MA, United States
AlexaFluor647-donkey-anti-goat IgG (A21447)	1:250	Thermo Fisher Scientific, Waltham, MA, United States
AlexaFluor647-donkey-anti-rabbit IgG (A31573)	1:250	Thermo Fisher Scientific, Waltham, MA, United States
AlexaFluor647-donkey-anti-mouse IgG (A31571)	1:250	Thermo Fisher Scientific, Waltham, MA, United States
AlexaFluor488-donkey-anti-mouse IgG (A21202)	1:250	Thermo Fisher Scientific, Waltham, MA, United States
**Western blot**		
**Primary antibodies**		
Guinea pig-anti-CNP-1 (polyclonal, 355004)	1:4,000	Synaptic Systems, Göttingen, Germany
Rabbit-anti-MBP (polyclonal, 295002)	1:1,500	Synaptic Systems, Göttingen, Germany
Rabbit-anti-Laminin (polyclonal, NB300-144)	1:500	Novus, Littleton, CO, United States
Mouse-anti-β-Actin 8H10D10 (monoclonal, 3700S)	1:1,500	Cell Signaling Technology, Danvers, MA, United States
**Secondary antibodies**		
HRP-rabbit-anti-guinea pig IgG	1:2,000	Dako Denmark A/S, Glostrup, Denmark
HRP-goat-anti-rabbit IgG	1:10,000	Vector Laboratories., Burlingame, CA, United States
HRP-horse-anti-mouse IgG	1:10,000	Vector Laboratories., Burlingame, CA, United States

### Immunofluorescence microscopy and intensity quantification

**FIGURE 1:**
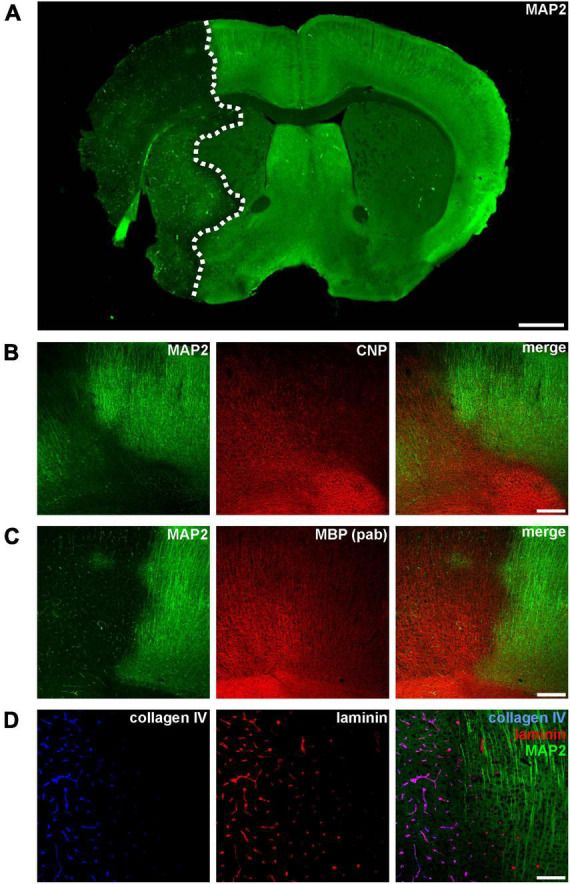
**(A)** Immunofluorescence labeling of MAP2 in a mouse forebrain after 24 h of MCAO. Due to its sensitivity to ischemia, a reduction of MAP2-related immunoreactivity was used to detect the ischemic infarct area (outlined with a dashed line). **(B–D)** Micrographs depict the increased immunofluorescence labeling of CNP, MBP, collagen IV, and laminin in the ischemic infarct area at the cortical border of the infarct (infarct on the left side). Scale bars: **(A)** 1 mm; **(B,C)** 200 μm; **(D)** 100 μm.

After immunofluorescence labeling, the sections were scanned in an Axio Scan.Z1 slide scanner (Carl Zeiss Microscopy GmbH, Jena, Germany) to quantify immunofluorescence intensity signals related to CNP, MBP, collagen IV, and laminin in two different sections per animal (*n* = 4–5). Here, the increase of the respective immunofluorescence signals in the ischemic region was correlated with the decreased immunofluorescence signal of the microtubule-associated protein 2 (MAP2), which is known to be very sensitive to ischemia (Kitagawa et al., 1989; Dawson and Hallenbeck, 1996; Popp et al., 2009), thereby confirming that the measurements are limited to the area of infarction (Figure 1). Thus, regions of interest (ROIs) were placed in ischemia-affected areas of high immunofluorescence reactivity in the cortex and in the myelinated fiber tracts of the striatum and were accordingly mirrored to the unaffected contralateral hemisphere. For additional quantifications of laminin-related fluorescence intensity in 10 μm-thick fresh-frozen sections and trypsin-treated sections (Figure 5), vessels were captured using a 10 × magnification throughout the ipsi- and contralateral hemisphere. Tissue-specific background was excluded per threshold and vessels were quantified using the particle analysis. Thus, for each animal the mean fluorescence intensity of a minimum of 64 vessels per hemisphere was measured (*n* = 4–5).

In general, mean values were generated per region and animal while the mean values of the contralateral regions (cortex or striatum) served as control. The n-fold change was then calculated as the ratio of the mean value per animal and region and the respective control value. Image exports were performed using the netScope Viewer software (Net-base Software GmbH, Freiburg im Breisgau, Germany) and the fluorescence intensity was measured using the Image J software (National Institutes of Health, Bethesda, MD, USA).

### Western blots

Mice used for Western blots were perfused with saline, only. The brains were removed and the area of the infarct was macroscopically identified by its tissue edema, then manually dissected and the resulting samples (cortex and striatum of each hemisphere) were snap-frozen in liquid nitrogen and stored at −80°C. Each brain sample was homogenized and lysed by ultra-sonification on ice in a solution of 60 mM Tris–HCl, pH 6.8, which contained 10% sucrose, 2% sodium dodecyl sulfate (SDS), and a protease inhibitor cocktail (Cell Signaling, Leiden, Netherlands), followed by centrifugation (13,000 rpm, 4°C, 10 min).

To measure the protein concentration, the BCA kit (Thermo Fisher Scientific, Waltham, MA, USA) was used. Protein denaturation was performed in sample buffer consisting of 250 mM Tris–HCl (pH 6.8), which contains 4% SDS, 10% glycerol as well as 2% β-mercaptoethanol, at a temperature of 95°C for 5 min. Afterward, samples were quickly centrifuged again. To separate the proteins a 4–20% SDS-PAGE was used for Western blotting of MBP and CNP and a 12% SDS-PAGE for laminin. Then, proteins were transferred onto a nitrocellulose membrane (Th. Geyer, Renningen, Germany) by blotting at 25 V. To control sample loading, a total protein staining using 0.1% Ponceau S (Sigma, Steinheim, Germany) in 5% acetic acid was applied, then washed off again in washing buffer (6 g/l Tris, 8.8 g/l NaCl, 3 ml/l Tween 20). Subsequently, membranes were either blocked in a Roti-block solution (Carl Roth, Karlsruhe, Germany; 1:10 dilution in washing buffer) or in 5% dry milk in TBS (50 mM Tris–HCl, 150 mM NaCl, pH 7.5), for 30 min. Thereafter, they were incubated in a solution of primary antibodies (Table 1) at 4°C overnight. The next day, membranes were thoroughly rinsed with washing buffer and then incubated with horseradish peroxidase (HRP)-conjugated secondary antibodies (Table 1) for 1 h at room temperature. Membrane development and image acquisition was performed using the ECL kit (Thermo Fisher Scientific). In order to calculate relative protein concentrations of MBP, CNP and laminin, membranes were stripped with a stripping buffer (15 g/l glycine, 1 g/l SDS, 10 ml/l Tween 20, pH 2.2) and reused to detect β-actin as housekeeping protein for reference. The integrated optical densities (IOD) of the target proteins were quantified and normalized in relation to ß-actin. Then, mean values per animal and region were created, while contralateral regions (cortex or striatum) served as control. For each region, the n-fold change was calculated as the ratio between mean values of the ischemia-affected areas and the respective control region.

To rule out ischemia-mediated changes of ß-actin expression, the IOD values of target proteins were also normalized using a total protein stain with Ponceau S as recommended by Fosang and Colbran (2015). Here, lanes stained with Ponceau S (as described above) were quantified by totaling all band intensities. IODs of the target proteins were calculated as a ratio to the total protein value of the respective lane.

For MBP, measurements comprise the 14, 17, 18.5, and 21.5 kDa MBP isoforms. For laminin, measurements comprise the 220 kDa beta 1 and gamma 1 laminin subunits. The sample size was *n* = 4–5 for each analysis.

### Statistical analyses

For statistical data analysis GraphPad Prism 9.5v software (GraphPad Software Inc., La Jolla, CA, USA) was used. All data was subjected to the Grubbs’ test to check for statistical outliers followed by the non-parametric Mann-Whitney test to check for statistical significance. In general, a *p* < 0.05 was considered statistically significant.

## Results

### The fluorescence intensity related to CNP, MBP, collagen IV, and laminin is increased in areas of ischemia-derived MAP2 reduction

Ischemia-affected areas can be identified by an apparent reduction of MAP2 immunoreactivity (Dawson and Hallenbeck, 1996; Härtig et al., 2016; Mages et al., 2021). In addition, our group was able to identify several other markers to precisely identify ischemia-affected tissue in various models of MCAO, including the oligodendrocyte markers CNP and MBP as well as the vasculature associated basement membrane components laminin and collagen IV (Hawkes et al., 2013; Michalski et al., 2018, 2020; Mages et al., 2019). However, while ischemia-affected areas are characterized by a reduction of MAP2-related fluorescence intensity, an opposite effect as indicated by an increased intensity is observed for CNP, MBP, collagen IV, and laminin (Figure 1).

### CNP-related immunofluorescence intensity increases in ischemia-affected areas while an increase at the protein level was not confirmed by Western blot analysis

**FIGURE 2:**
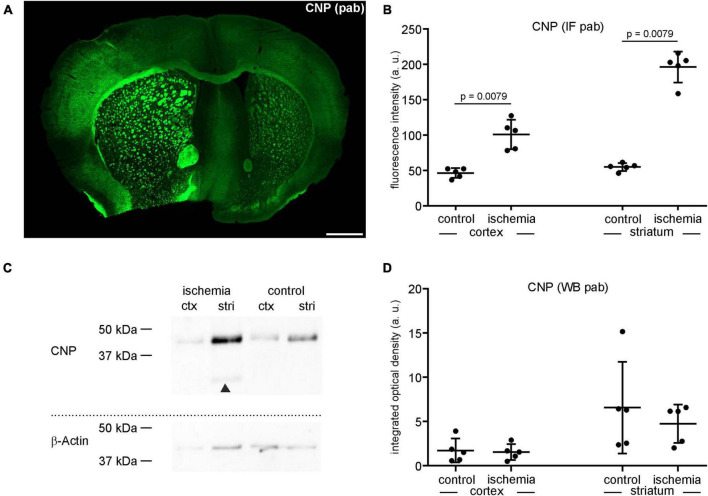
**(A)** Overview of an immunofluorescence labeling using a polyclonal (pab) anti-CNP antibody in a mouse forebrain section after MCAO (infarct on the left side). Scale bar: 1 mm. **(B)** Quantification of CNP-related immunofluorescence intensity (IF) in immunolabeled sections reveals a significant increase in ischemic tissue compared to contralateral control tissue (*n* = 5). **(C,D)** Western blot (WB) analysis reveals no increase of CNP in ischemic tissue. The CNP protein level is slightly lower in ischemic tissue than in control tissue (*n* = 5). Arrowheads: bands of lower molecular weight indicative of degradation products, ctx, cortex; stri, striatum. Data are given as mean values; error bars indicate SD.

Since the data on ischemia-related protein affection is found to be contradictory, we next investigated whether the visually increased immunoreactivity of the markers shown in Figure 1 is indeed indicative of increased protein levels. Therefore, CNP-related immunofluorescence intensity was measured in ischemia-affected areas, thereby confirming a 2.2-fold increase in the ischemic cortex and a 3.6-fold increase as compared to contralateral control areas (Figures 2A, B and Supplementary Table 1). However, despite using the same polyclonal antibody, this increase was not confirmed by Western blot analyses.

Here, the integrated optical density of ischemia-affected cortical and striatal areas equates to a fold-change of 0.9 in the cortex and 0.7 in the striatum, respectively (Figures 2C, D). Of note, CNP-derived degradation products of lower molecular weight were detectable in the ischemia-affected tissue (Figure 2C).

Importantly, for each of the analyzed target proteins the possibility of ischemia-derived changes of ß-actin expression was addressed by normalizing the target proteins to a Ponceau S total protein staining (Fosang and Colbran, 2015; Sander et al., 2019), which confirmed the results obtained by normalization for ß-actin (Supplementary Figure 2 and Supplementary Table 1).

### Increase of MBP-related fluorescence intensity using polyclonal antibodies cannot be confirmed by monoclonal antibodies or Western blot analysis

**FIGURE 3:**
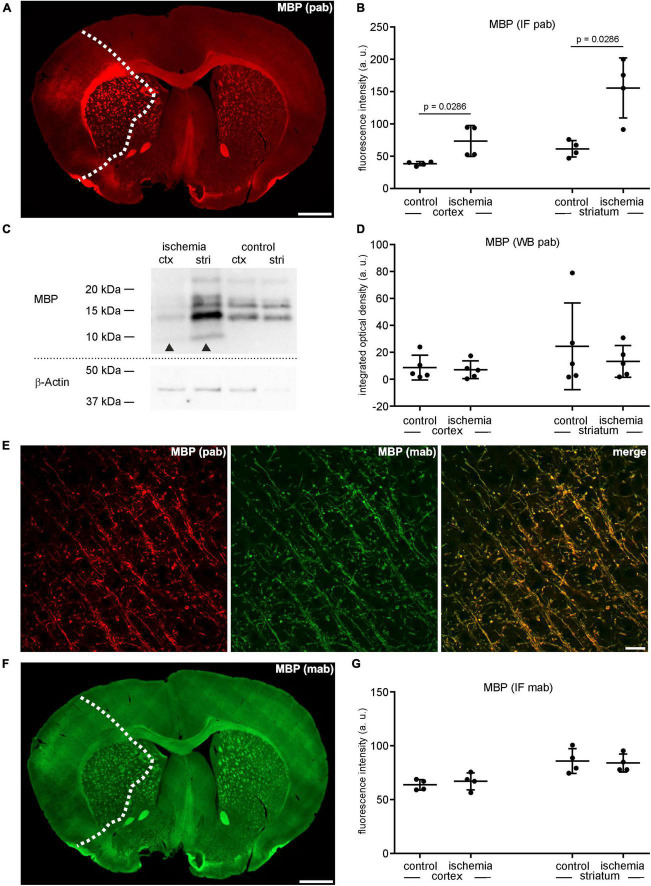
**(A)** Overview of an immunofluorescence labeling using a polyclonal (pab) anti-MBP antibody in a mouse forebrain section after MCAO (infarct on the left side; dashed line). **(B)** Quantification of immunofluorescence intensity in MBP-labeled sections using a polyclonal antibody reveals a significant increase in ischemic tissue compared to contralateral areas (*n* = 4). **(C,D)** Western blot analysis did not reveal increased MBP protein levels in ischemic tissue (*n* = 5). Arrowheads: bands of lower molecular weight indicative of degradation products, ctx, cortex; stri, striatum. **(E)** Confocal micrographs from the ischemic cortex show the specificity of the polyclonal and the monoclonal (mab) MBP antibody by its co-localization. **(F)** Overview of an immunofluorescence labeling using a monoclonal anti-MBP antibody in a mouse forebrain section after MCAO (infarct on the left side; dashed line). **(G)** Quantification of immunofluorescence intensity in MBP-labeled sections using a monoclonal antibody reveals an unchanged immunofluorescence intensity in ischemic and control tissue (*n* = 4). Data are given as mean values; error bars indicate SD. Scale bars: **(A,F)** 1 mm; **(E)** 20 μm.

Similar to CNP as outlined above, MBP was analyzed using the same polyclonal anti-MBP antibody for both immunofluorescence labeling and Western blot analysis. In line, the MBP-related immunofluorescence intensity was regularly increased in the ischemic tissue of cortical (1.9-fold change) and striatal (2.5-fold change) areas as well as in the adjacent corpus callosum (Figures 3A, B and Supplementary Table 1). However, contrary to the immunofluorescence labeling, Western blot analyses did not indicate an increase of MBP protein levels in the ischemic tissue, showing a fold-change of 0.81 in the ipsilateral cortex and 0.54 in the ipsilateral striatum compared to contralateral control tissue (Figures 3C, D and Supplementary Table 1). As shown for CNP, MBP-related degradation products of lower molecular weight were also detectable in ischemic tissue (Figure 3C). To investigate whether the described increase of MBP-related fluorescence intensity can also be demonstrated with applicable monoclonal antibodies, we performed double immunofluorescence labeling. Of note, application of the monoclonal antibody in combination with the polyclonal strictly demonstrated co-localizations of neuronal structures (Figure 3E). However, the immunofluorescence intensity related to the monoclonal antibody did not increase (ischemic cortex: 1.1-fold change; ischemic striatum 0.98-fold change), although the measurements were performed in the same ROIs used for the polyclonal antibody (Figures 3F, G and Supplementary Table 1). Importantly, omitting the polyclonal MBP antibody did not result in an increased immunoreactivity of the monoclonal antibody, either (Supplementary Figure 3). Thus, the possibility, that simultaneous labeling with the polyclonal antibody blocks binding sites for the monoclonal one, was ruled out.

### The vascular markers collagen IV and laminin show increased immunofluorescence intensities while increased protein levels of laminin were not detected by Western blot analysis

**FIGURE 4:**
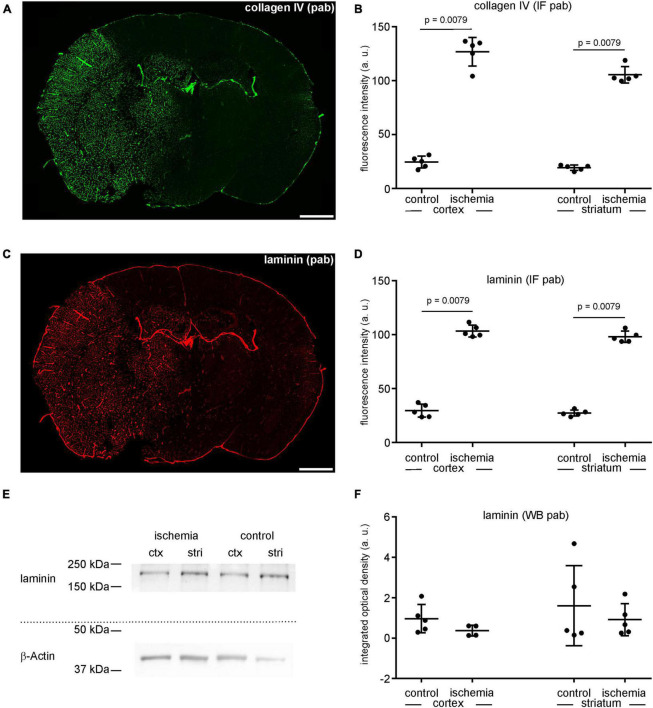
**(A)** Overview of an immunofluorescence labeling using a polyclonal (pab) anti-collagen IV antibody in a mouse forebrain section after MCAO (infarct on the left side). **(B)** Quantification of immunofluorescence intensity in collagen IV-labeled sections using a polyclonal antibody reveals a significantly increased immunofluorescence intensity in ischemic tissue (*n* = 5). **(C)** Overview of an immunofluorescence labeling using a polyclonal anti-laminin antibody in a mouse forebrain section after MCAO (infarct on the left side). **(D)** Quantification of immunofluorescence intensity in laminin-labeled sections using a polyclonal antibody reveals a significant increase in ischemic tissue compared to contralateral areas (*n* = 5). **(E,F)** Western blot analysis did not reveal increased laminin protein levels in ischemic tissue (*n* = 5). Ctx, cortex; stri, striatum. Data are given as mean values; error bars indicate SD. Scale bars: 1 mm.

As described above, the immunofluorescence intensity related to the vascular basement membrane proteins laminin and collagen IV is increased in ischemia-affected areas (Figures 1, 4A, C). Labeling with polyclonal anti-collagen IV antibodies leads to a 5.2-fold increase in the ischemic cortex and a 5.3-fold increase in the ischemic striatum compared to contralateral control tissue (Figures 4A, B and Supplementary Table 1). The same pattern can be observed when applying polyclonal antibodies to laminin. Here, the immunofluorescence intensity is increased 3.5-fold in the ischemia-affected cortex and 3.6-fold in the striatum (Figures 4C, D and Supplementary Table 1). Again, while laminin-related immunofluorescence intensities appear to be increased in the infarcted areas, Western blot analysis did not indicate increased protein levels in the ischemic cortical (0.4-fold change) and striatal (0.6-fold change) areas compared to controls (Figures 4E, F and Supplementary Table 1).

### Tissue processing and pre-treatment influences immunofluorescence intensities of laminin

**FIGURE 5:**
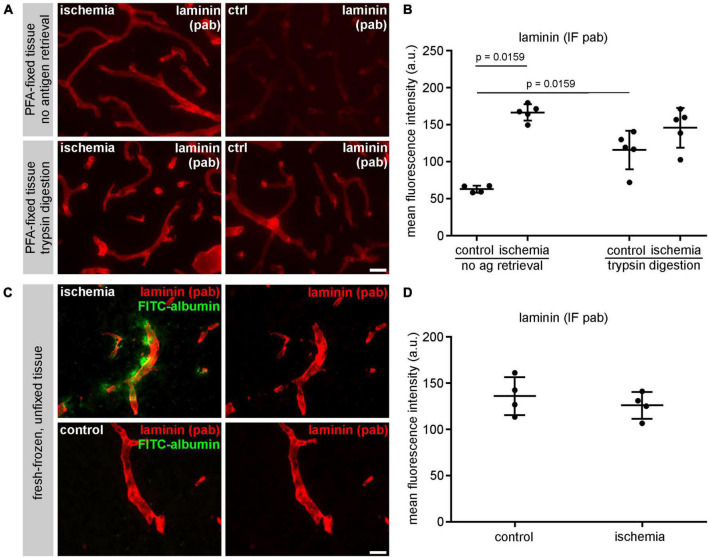
**(A)** The immunofluorescence intensity using a polyclonal (pab) anti-laminin antibody is higher in non-ischemic control tissue after antigen retrieval with trypsin digestion. **(B)** Quantification of untreated and trypsin-treated sections reveals a significant increase of laminin-related immunofluorescence intensity in vessels of the contralateral hemisphere after trypsin digestion (*n* = 5). **(C)** Immunofluorescence labeling using a polyclonal anti-laminin antibody in fresh-frozen, unfixed tissue (without PFA-fixation). Paravascular FITC-albumin signal reveals areas of BBB breakdown and thus ischemic infarct tissue. **(D)** Quantification of unfixed laminin-labeled tissue sections reveals similar immunofluorescence intensities of vessels in ischemic and control tissue (*n* = 4). Data are given as mean values; error bars indicate SD. Scale bars: **(A)** 20 μm; **(C)** 20 μm.

In order to address the impact of methodological differences on immunofluorescence intensity measurements, brain immunofluorescence labeling of laminin using the same polyclonal antibody was applied after different pre-treatments. First, the same tissue used for immunofluorescence analysis of laminin as shown in Figure 4 was pretreated with an enzymatic trypsin digestion, followed by labeling and quantification as described above. Here, the mean fluorescence intensity of contralateral control regions was increased (115.7 a.U.) when compared to untreated sections (62.9 a.u.) (Figures 5A, B and Supplementary Table 1). Of note, this increase was not apparent in ischemia-affected areas, where a difference between trypsin-treated and untreated sections was not observed. In a second approach, we used snap-frozen, unfixed tissue of the same mouse model, in which ischemia-affected areas were demarcated by extravasation of intravenously applied FITC-albumin. Prior to immunolabeling, the sections were fixed with ethanol only, followed by immunolabeling of laminin according to the same protocol. Of note, these sections did not exhibit the ischemia-derived increase of laminin-related fluorescence intensity (Figures 5C, D), which was regularly observed in PFA-fixed sections (Figure 4).

## Discussion

In the setting of ischemic stroke, several components of the NVU are believed to play a fundamental role in the transformation of reversible to irreversible tissue damage including the myelin-associated proteins CNP and MBP as well as the components of vascular basement membranes laminin and collagen IV (Ogata et al., 1989; Dewar and Dawson, 1997; Yuan et al., 2012; Mages et al., 2021). However, there is controversial data on the ischemia-mediated effects on the mentioned proteins, which are unlikely to be explained by differences in experimental setups alone.

Therefore, we used the widely applied model of filament-based MCAO, which offers the advantage of very high reproducibility and consistent infarct sizes (Durukan and Tatlisumak, 2007; Michalski et al., 2013; Sommer, 2017) to address whether the choice of antibody clonality and tissue pre-treatment impact on immunofluorescence intensity measurements. Further, we investigated, whether the obtained results actually correlate with protein levels as given by Western blot analyses. For this purpose, the tissue was analyzed 24 h after ischemia induction, which not only represents one of the most often applied time points in preclinical stroke research, but also reflects the clinical situation, where efforts are made to prolong the therapeutic time frame for concerned patients (Nair et al., 2023).

Using immunofluorescence microscopy, the loss of MAP2-related immunoreactivity allows a clear-cut detection of the ischemia-affected regions (Inuzuka et al., 1990; Vanicky et al., 1995; Mages et al., 2021). Strikingly, however, immunolabeling with polyclonal antibodies consistently revealed increased fluorescence intensities in these regions for CNP, MBP, laminin, and collagen IV (Figures 1, 2A, B, 3A, B, 4A–D) even though Western blot analyses did not indicate increased protein levels (Figures 2C, D, 3C, D, 4E, F).

The here reported discrepancy is in line with several reports in the literature. Here, Western blot analyses of a MCAO rat model also revealed a trend toward decreased CNP levels relative to controls after 24 h of ischemia, while statistical significance was reached after 3 days of MCAO (Inuzuka et al., 1990). Similar results are reported for MBP, either describing a reduction of MBP at the protein level or insignificant changes 24 h after ischemic onset (Chu et al., 2012; Buscemi et al., 2019; Zalewska et al., 2021). In contrast, studies using immunofluorescence labeling describe a post-ischemic increase of CNP and MBP-related signals (Michalski et al., 2018, 2022; Mages et al., 2019, 2021). The available literature regarding post-ischemic levels of laminin and collagen IV is also indeterminate. Western blot analyses revealed decreased protein levels of the two basement membrane proteins (Hamann et al., 2002; Zalewska et al., 2003; Vosko et al., 2006), while increased levels were also observed (Ji and Tsirka, 2012). This also accounts for studies using immunofluorescence microscopy where several studies describe increased collagen IV- and laminin-related intensities in different animal models at various time points (Anik et al., 2011; Ji and Tsirka, 2012; Hawkes et al., 2013; Härtig et al., 2017; Michalski et al., 2017, 2018, 2020), but also decreased intensities (Hamann et al., 1995, 2002; Gu et al., 2005; Vosko et al., 2006; McColl et al., 2008). Importantly, the majority of the above mentioned studies utilized comparable models of transient as well as permanent MCAO with time points mostly focusing on 24 h after ischemia induction.

Although, methodological differences of ischemia induction and observation time points should always be considered, these aspects alone are unlikely to explain the described contradictions. On the contrary, increased immunofluorescence intensities of CNP and collagen IV have been consistently observed using polyclonal antibodies in various models of ischemia and human autoptic brain tissue over different time points (Härtig et al., 2017; Mages et al., 2018, 2019, 2021; Michalski et al., 2020, 2022).

Of note, our group has previously demonstrated that the results obtained from fluorescence microscopy measurements also depend on the clonality of the applied antibody (Mages et al., 2018). Specifically, using a monoclonal neurofilament light chain (NF-L) antibody resulted in a decreased intensity, while a polyclonal NF-L antibody showed an increased intensity in ischemic tissue. However, Western blots revealed that the increased intensity is likely to result from a binding of the polyclonal antibody to NF-L degradation products, which were increased in the ischemic tissue, although the full-length NF-L protein was in fact diminished. In this context, the presence of degradation products can be attributed to proteolytic processes in ischemic brain tissue, as reported by previous studies (Rosenberg et al., 1996; Lee et al., 2004; Lo et al., 2004).

In the present study, Western blot analyses also revealed bands of lower molecular weight indicating the presence of respective degradation products of CNP and MBP in ischemic tissue (Figures 2, 3). Therefore, the enhanced immunoreactivity of CNP and MBP is likely to result from an increased binding of the polyclonal antibody to multiple epitopes, which become available by the presence of the respective degradation products. However, the levels of the full-length protein can remain unaltered or even decreased.

To our knowledge, there are no other studies so far demonstrating a correlation between increased intensity measurements in fluorescence microscopy and the abundance of degradation products. However, other studies have shown the presence of smaller protein fragments in various models, including NF-L (Posmantur et al., 1994) and MBP in models of traumatic brain injury and neurodegenerative autoimmunity (Liu et al., 2006; Belogurov et al., 2015), as well collagen IV (McColl et al., 2008) and laminin (Zalewska et al., 2003) in models of cerebral ischemia. In the setting of stroke, the principle of enzymatic degradation of laminin and collagen IV has further been shown to impact on basement membrane integrity and therefore on BBB function (Yao, 2019). Importantly, we also demonstrated that enzymatic digestion can enhance immunofluorescence intensity of polyclonal antibodies, as shown for laminin. Here, proteolytic antigen retrieval did not alter the general immunoreactivity on the sections *per se*. Instead, immunofluorescence intensity was mainly enhanced in non-ischemic areas, which were not already affected by ischemia-derived proteolytic alterations (Figures 5A, B).

Further, differences of paraformaldehyde-mediated effects on the ischemic tissue and non-affected control areas may explain the observed differences of immunofluorescence intensity in the analyzed sections. In general, formaldehyde fixation results in a variable loss of immunoreactivity by inducing the formation of cross-links between different parts of the antigen or between two or more molecules. These cross-links hamper the access of antibodies to the epitope and reduce the availability of reactive sites. Although some epitopes might be demasked by antigen retrieval methods such as enzyme digestion, for some epitopes the duration of the formaldehyde fixation is critical (Werner et al., 1996, 2000). It is therefore possible that the effect of epitope masking is weaker in the ischemic tissue of the brain, where permeation of the tissue with the fixative could be less effective during the transcardial perfusion due to the vessel occlusion. In addition, autolysis is more likely to occur in the ischemic tissue until immersion by the fixative is complete.

To address these effects, we used the same polyclonal antibody labeling procedure on fresh-frozen tissue without formaldehyde fixation. Prior to immunolabeling, sections were fixed with ethanol, only. Of note, with this approach the described increase of laminin-related immunofluorescence intensity in ischemia-affected areas was not detectable anymore (Figures 5C, D). This suggests that paraformaldehyde-mediated effects on epitope masking and antigenicity can indeed differ when comparing ischemia-affected and non-affected control tissue and thus one-sidedly impact on immunofluorescence intensity measurements. Vice versa, these effects could be avoided by snap-freezing of the tissue and post-fixation of cryostat sections prior to immunolabeling.

In summary, low epitope masking, proteolysis and autolysis are possible factors which contribute to a higher abundance of degradation products and multiple epitopes thereby resulting in a higher immunoreactivity of polyclonal antibodies in the ischemic tissue. Therefore, this problem could be avoided by using monoclonal antibodies, which are only specific for a single epitope. This is supported by the results obtained from MBP labeling with monoclonal antibodies, which did not result in an increased intensity in the ischemic tissue (Figures 3E–G). Unfortunately, sufficient labeling with monoclonal antibodies can be challenging and may require antigen retrieval, which is not always practicable. Since infarcted brain tissue already exhibits a reduced mechanical integrity (Michalski et al., 2015; Mages et al., 2021), enzymatic digestion or heating cannot be applied for certain tissue preparations. For these reasons and despite great efforts, we were not able to establish a reliable immunolabeling for CNP, collagen IV, and laminin by monoclonal antibodies in this manuscript.

Furthermore, monoclonal antibodies are often mouse-derived and respective secondary anti-mouse antibodies can be disadvantageous on mouse tissue. Since MCAO naturally hinders perfusion of the vessels in the ischemic territory after the sacrifice, remaining intrinsic mouse antibodies may increase the risk of unspecific labeling with secondary anti-mouse antibodies in the ischemic area. Unfortunately, this applies to many preclinical stroke experiments, as the majority of stroke models are performed in mice and rats (Durukan and Tatlisumak, 2007; Braeuninger and Kleinschnitz, 2009; Fluri et al., 2015). Although other host species for monoclonal antibodies might be an alternative, the availability is often limited as rodents still represent the most common host. This may explain, why analyses based on immunolabeling in stroke tissue are mostly performed using polyclonal antibodies (CNP: Jing et al., 2013; Michalski et al., 2018; Mages et al., 2019, 2021; MBP: Gregersen et al., 2001; Zhao et al., 2014; Michalski et al., 2018; collagen IV: Hamann et al., 2002; Vosko et al., 2006; Anik et al., 2011; Ji and Tsirka, 2012; Härtig et al., 2017; Michalski et al., 2017, 2020, 2022; laminin: Wagner et al., 1997; Gu et al., 2005; Ji and Tsirka, 2012).

In conclusion, it is improbable that the here shown increased immunoreactivities of CNP, MBP, collagen IV, and laminin reflect an actual increase at the protein level. They are more likely to reflect an increased antibody-related immunoreactivity due to an increase of degradation products and higher availability of reactive binding sites. Furthermore, this early after ischemia induction, an increased protein expression is also unlikely due to different processes contributing to ischemia-induced protein synthesis suppression (Hossmann, 1993; Mengesdorf et al., 2002; Kokubo et al., 2003; Zhang et al., 2006) or simply due to ischemic cell death (Lipton, 1999; Dirnagl, 2012; Zille et al., 2012). However, the authors acknowledge that this study does not address possible functional or time-dependent changes after stroke, but focuses on methodical aspects instead.

Overall, the here applied analyses emphasize how tissue processing, pre-treatment and antibody clonality can impact on the reproducibility of immunofluorescence analyses, especially in ischemic stroke tissue. Although immunofluorescence labeling represents an elegant tool to capture the localization or distribution of proteins throughout the tissue or in cellular compartments, fluorescence intensity measurements do not necessarily mirror the actual protein levels. Therefore, it is mandatory to interpret data obtained by immunofluorescence intensity measurements of ischemic tissue with caution and to validate the results with different methods. Thereby, our understanding of ischemia-mediated effects on the NVU will hopefully be facilitated and the reproducibility enhanced.

## Data availability statement

The raw data supporting the conclusions of this article will be made available by the authors, without undue reservation.

## Ethics statement

This animal study was reviewed and approved by the Landesdirektion Sachsen, Leipzig, Germany.

## Author contributions

BF and MK: study design. AP, BF, and MK: manuscript and figures preparation. BF: statistical analysis. DM, MK, and BF: animal experiments and associated regulatory affairs. AP: immunofluorescence labeling and microscopy. AP and CH: western blot analysis. WH, DM, MK, and BF: critical revision. All authors contributed to the article and approved the submitted version.

## Supplementary material

Supplementary Figure 1 Representative images showing negative controls. Primary antibodies were omitted. Thereby, application of the secondary AlexaFluor (AF) conjugated donkey (dk) antibodies (as listed in Table 1) resulted in absence of labeling. Scale bar: 50 μm.

Supplementary Figure 2 Western blot analyses of **(A)** CNP (*n* = 5), **(C)** MBP (*n* = 5), and **(E)** laminin (*n* = 4) are shown after normalization using a total protein staining with Ponceau S **(B,D,F)**. The data confirmed the results obtained by normalization to ß-actin as shown in Figures 2–4. Ctx, cortex; stri, striatum. Data are given as mean values; error bars indicate SD.

Supplementary Figure 3 Overview of an immunofluorescence labeling using a monoclonal (mab) anti-MBP antibody in a mouse cortex after MCAO (infarct on the left side; dashed line). Simultaneous labeling with the polyclonal (pab) anti MBP-antibody was omitted to rule out a potential blocking of binding sites. Still, the MBP (mab) immunoreactivity remained unchanged in ischemic areas, which are demarcated by a decrease of MAP2- and an increase of collagen IV-related immunofluorescence intensity. Scale bar: 200 μm.
