# Supplementary material for: Increasing reproducibility in preclinical stroke research: the correlation of immunofluorescence intensity measurements and Western blot analyses strongly depends on antibody clonality and tissue pre-treatment in a mouse model of focal cerebral ischemia
Source: Front Cell Neurosci. 2023 Jun 2;17:1183232. doi: 10.3389/fncel.2023.1183232 (PMC10277931; doi:10.3389/fncel.2023.1183232)
Supplement: Supplementary file 1 [file Table_1.DOCX]

| **figure** | **analysis** | **dataset** | | **n=** | **mean** | **median** | **SD** | **MD** | **fold-change** | **p-value** |
| --- | --- | --- | --- | --- | --- | --- | --- | --- | --- | --- |
| 2b | **CNP**  **IF (pab)** | cortex | control | 5 | 46.56 | 48.48 | 6.89 | 54.34 | **2.17** | 0.0079 |
|  |  |  | ischemia | 5 | 100.90 | 106.80 | 20.87 |  |  |  |
|  |  | striatum | control | 5 | 55.14 | 55.97 | 5.61 | 141.16 | **3.56** | 0.0079 |
|  |  |  | ischemia | 5 | 196.30 | 203.10 | 21.93 |  |  |  |
| 2d | **CNP**  **WB (pab)** | cortex | control | 5 | 1.72 | 1.52 | 1.35 | 0.16 | **0.91** | ns |
|  |  |  | ischemia | 5 | 1.56 | 1.56 | 0.89 |  |  |  |
|  |  | striatum | control | 5 | 6.57 | 6.31 | 5.19 | 1.83 | **0.72** | ns |
|  |  |  | ischemia | 5 | 4.75 | 6.16 | 2.17 |  |  |  |
| 3b | **MBP**  **IF (pab)** | cortex | control | 4 | 38.56 | 39.20 | 3.12 | 35.10 | **1.91** | 0.0286 |
|  |  |  | ischemia | 4 | 73.66 | 73.55 | 24.06 |  |  |  |
|  |  | striatum | control | 4 | 61.63 | 61.60 | 12.76 | 94.07 | **2.53** | 0.0286 |
|  |  |  | ischemia | 4 | 155.70 | 165.60 | 46.49 |  |  |  |
| 3d | **MBP**  **WB (pab)** | cortex | control | 5 | 8.65 | 4.18 | 9.17 | 1.68 | **0.81** | ns |
|  |  |  | ischemia | 5 | 6.97 | 6.79 | 6.60 |  |  |  |
|  |  | striatum | control | 5 | 24.35 | 11.46 | 32.18 | 11.13 | **0.54** | ns |
|  |  |  | ischemia | 5 | 13.22 | 11.65 | 11.78 |  |  |  |
| 3g | **MBP**  **IF (mab)** | cortex | control | 4 | 63.78 | 63.52 | 4.93 | 3.24 | **1.05** | ns |
|  |  |  | ischemia | 4 | 67.02 | 68.00 | 7.88 |  |  |  |
|  |  | striatum | control | 4 | 85.76 | 83.97 | 11.47 | 1.83 | **0.98** | ns |
|  |  |  | ischemia | 4 | 83.93 | 81.33 | 8.28 |  |  |  |
| 4b | **collagen IV**  **IF (pab)** | cortex | control | 5 | 24.52 | 25.25 | 5.50 | 102.28 | **5.17** | 0.0079 |
|  |  |  | ischemia | 5 | 126.80 | 132.70 | 13.30 |  |  |  |
|  |  | striatum | control | 5 | 19.33 | 20.44 | 2.51 | 86.27 | **5.46** | 0.0079 |
|  |  |  | ischemia | 5 | 105.60 | 102.60 | 7.68 |  |  |  |
| 4d | **laminin**  **IF (pab)** | cortex | control | 5 | 29.58 | 28.33 | 6.05 | 73.82 | **3.50** | 0.0079 |
|  |  |  | ischemia | 5 | 103.40 | 101.00 | 5.55 |  |  |  |
|  |  | striatum | control | 5 | 27.37 | 26.73 | 2.84 | 70.70 | **3.58** | 0.0079 |
|  |  |  | ischemia | 5 | 98.07 | 96.59 | 5.21 |  |  |  |
| 4f | **laminin**  **WB (pab)** | cortex | control | 5 | 0.97 | 0.89 | 0.70 | 0.59 | **0.39** | ns |
|  |  |  | ischemia | 4 | 0.38 | 0.38 | 0.27 |  |  |  |
|  |  | striatum | control | 5 | 1.60 | 0.38 | 1.98 | 0.68 | **0.57** | ns |
|  |  |  | ischemia | 5 | 0.92 | 0.67 | 0.79 |  |  |  |
| 5b | **laminin**  **IF (pab)** | no ag retr | control | 4 | 62.89 | 63.00 | 4.80 | 103.51 | **2.65** | 0.0159 |
|  |  |  | ischemia | 5 | 166.40 | 167.70 | 11.03 |  |  |  |
|  |  | trypsin | control | 5 | 115.70 | 119.20 | 26.13 | 30.10 | **1.26** | ns |
|  |  |  | ischemia | 5 | 145.80 | 156.70 | 26.80 |  |  |  |
| 5d | **laminin**  **IF (pab)** | f-f, unfixed | control | 4 | 135.90 | 134.50 | 20.53 | 10.00 | **0.93** | ns |
|  |  |  | ischemia | 4 | 125.90 | 128.00 | 14.43 |  |  |  |
| Suppl. 2 a | **CNP**  **WB (pab)**  **TPN** | cortex | control | 5 | 0.0256 | 0.0287 | 0.0120 | 0.0013 | **1.05** | ns |
|  |  |  | ischemia | 5 | 0.0268 | 0.0301 | 0.0081 |  |  |  |
|  |  | striatum | control | 5 | 0.1000 | 0.0996 | 0.0312 | 0.0119 | **0.88** | ns |
|  |  |  | ischemia | 5 | 0.0881 | 0.0948 | 0.0232 |  |  |  |
| Suppl. 2 c | **MBP**  **WB (pab)**  **TPN** | cortex | control | 5 | 0.0581 | 0.0509 | 0.0354 | 0.0048 | **0.92** | ns |
|  |  |  | ischemia | 5 | 0.0533 | 0.0543 | 0.0296 |  |  |  |
|  |  | striatum | control | 5 | 0.1943 | 0.1987 | 0.0512 | 0.0173 | **0.91** | ns |
|  |  |  | ischemia | 4 | 0.1770 | 0.1821 | 0.0532 |  |  |  |
| Suppl. 2 e | **laminin**  **WB (pab)**  **TPN** | cortex | control | 4 | 0.0256 | 0.0293 | 0.0099 | 0.0011 | **1.04** | ns |
|  |  |  | ischemia | 4 | 0.0268 | 0.0241 | 0.0113 |  |  |  |
|  |  | striatum | control | 4 | 0.0349 | 0.0373 | 0.0267 | 0.0113 | **0.68** | ns |
|  |  |  | ischemia | 4 | 0.0236 | 0.0223 | 0.0206 |  |  |  |

**Supplementary table 1:**

Datasets of all analyses performed in this study. CNP = 2',3'-cyclic-nucleotide 3'-phosphodiesterase; MBP = myelin basic protein; IF = immunofluorescence; WB = Western blot; pab = polyclonal antibody; mab = monoclonal antibody; TPN = total protein normalization; no ag retr = no antigen retrieval; trypsin = trypsin digestion; f-f = fresh-frozen; SD = standard deviation of the mean; MD = difference in means; fold-change = fold-change of control (mean of ischemia / mean of control); p-value = p-value of ischemia vs. control (Mann-Whitney-U-test); ns= not significant (p>0.05). Values are given in arbitrary units.
