# Supplementary figures and images for: Increasing reproducibility in preclinical stroke research: the correlation of immunofluorescence intensity measurements and Western blot analyses strongly depends on antibody clonality and tissue pre-treatment in a mouse model of focal cerebral ischemia
Source: Front Cell Neurosci. 2023 Jun 2;17:1183232. doi: 10.3389/fncel.2023.1183232 (PMC10277931; doi:10.3389/fncel.2023.1183232)

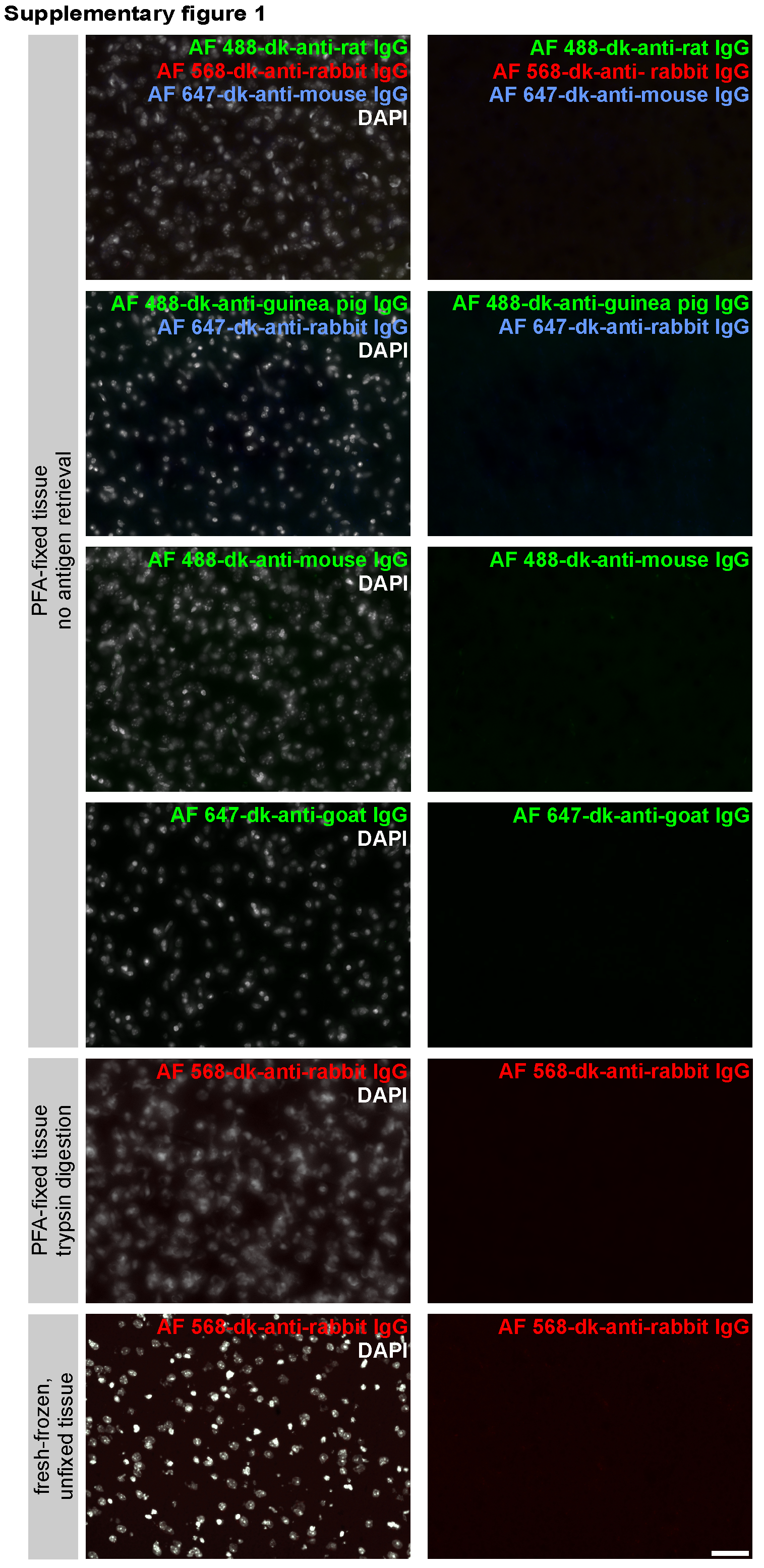

Supplement: Supplementary Figure 1 — Representative images showing negative controls. Primary antibodies were omitted. Thereby, application of the secondary AlexaFluor (AF) conjugated donkey (dk) antibodies (as listed in Table 1) resulted in absence of labeling. Scale bar: 50 μm. [file Image_1.TIF]

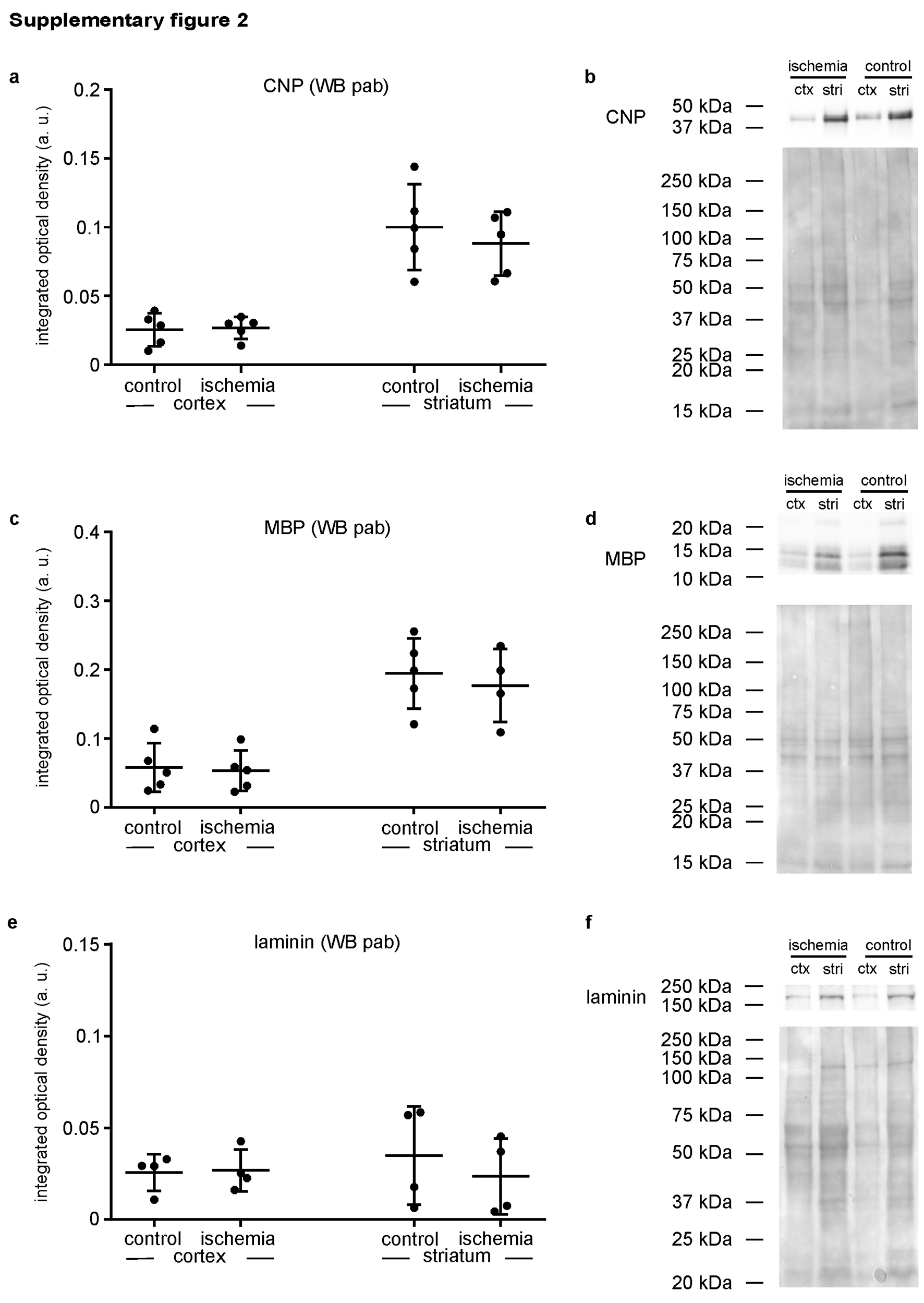

Supplement: Supplementary Figure 2 — Western blot analyses of (A) CNP (n = 5), (C) MBP (n = 5), and (E) laminin (n = 4) are shown after normalization using a total protein staining with Ponceau S (B,D,F). The data confirmed the results obtained by normalization to ß-actin as shown in Figures 2–4. Ctx, cortex; stri, striatum. Data are given as mean values; error bars indicate SD. [file Image_2.TIF]

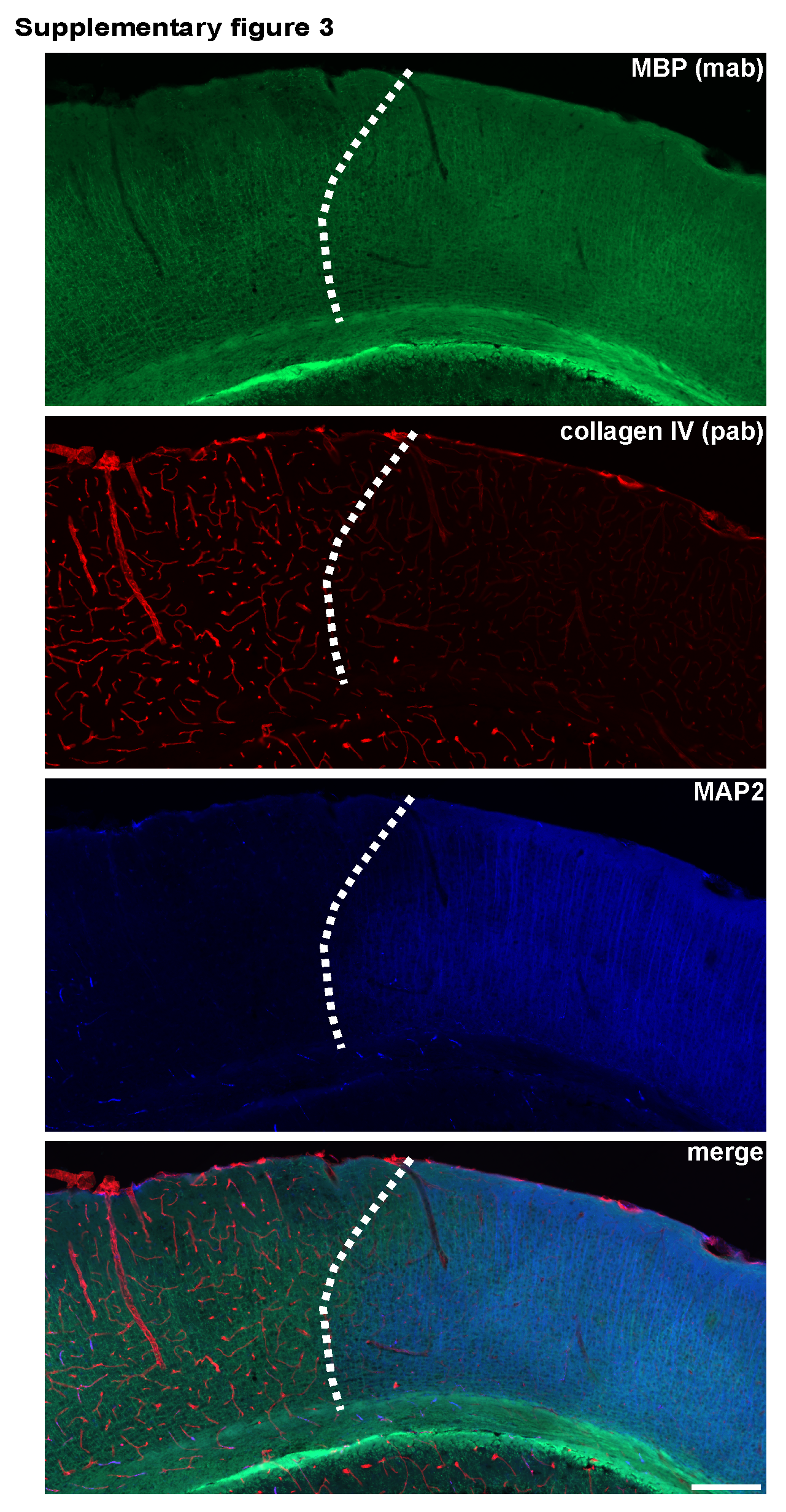

Supplement: Supplementary Figure 3 — Overview of an immunofluorescence labeling using a monoclonal (mab) anti-MBP antibody in a mouse cortex after MCAO (infarct on the left side; dashed line). Simultaneous labeling with the polyclonal (pab) anti MBP-antibody was omitted to rule out a potential blocking of binding sites. Still, the MBP (mab) immunoreactivity remained unchanged in ischemic areas, which are demarcated by a decrease of MAP2- and an increase of collagen IV-related immunofluorescence intensity. Scale bar: 200 μm. [file Image_3.TIF]
